# Efficacy of a novel oxygen scavenger mask in reducing local oxygen concentrations below the surgical fire risk threshold: an experimental proof-of-concept study

**DOI:** 10.1186/s13037-024-00411-1

**Published:** 2024-09-12

**Authors:** Christopher D. Yang, Teresa H. Chen, Jeremiah P. Tao

**Affiliations:** 1 Division of Oculofacial and Orbital Surgery, Gavin Herbert Eye Institute, Irvine, CA USA; 2 UC Irvine Health, Irvine, CA USA; 3 University of California Irvine School of Medicine, Irvine, CA USA

**Keywords:** Facial surgery, Operating room safety, Fire risk reduction, Operating room fires

## Abstract

**Background:**

This study aims to evaluate the efficacy of an oxygen scavenging mask device in reducing local oxygen concentrations from nasal cannula ventilation compared to a standard open facial surgical field.

**Methods:**

This is a controlled experiment using a custom-fabricated silicone midfacial oxygen scavenging device, SimMan airway management trainer manikin (Laerdal Medical, Stavanger, Norway), handheld oxygen detector (Forensics Detectors, Los Angeles, United States) and oxygen from a Datex Ohmeda Aisys Carestation anesthesia unit (GE HealthCare, Chicago, United States). Oxygen concentrations were measured at 18 facial landmarks (*Fig. 1*) with nasal cannula flow of 2, 4, and 6 L/min of 100% FiO2 in both masked and unmasked conditions (*Fig. 2*).

**Results:**

The mean oxygen concentration in the facial surgical field was 20.95% with the scavenger mask and 24.8% without (*P* < 0.001; two-tailed paired t-test). The unmasked condition was associated with suprathreshold oxygen concentration levels at 13 of 18 facial landmarks (*Table 1*). The device significantly reduced local oxygen concentration at 16 of 18 facial landmarks (*Table 1*). The device provided safe oxygen concentration levels at all three flow rates, and measured oxygen concentrations directly correlated with oxygen flow rate in the unmasked condition (*Table 2*).

**Conclusions:**

An oxygen scavenger mask device reduced local oxygen concentrations from nasal cannula ventilation to below the 23% fire threshold in the entire facial surgical field external to the mask in these experiments. The device may reduce intraoperative fire risk in patients that require supplementary oxygen during surgery.

**Supplementary Information:**

The online version contains supplementary material available at 10.1186/s13037-024-00411-1.

## Background

Intraoperative surgical fires represent a substantial health burden. Despite public awareness and implementation of measures to prevent surgical fires, many still occur each year, primarily in procedures involving the head or neck [1]. Although drastically underreported, the Food and Drug Administration Manufacturer and User Facility Device Experience (MAUDE) database reports at least 565 surgical fire events in the United States causing patient or staff harm during the years 2000 to 2020 [2]. Emergency Care Research Institute (ECRI), an independent non-profit organization dedicated to improving healthcare safety, estimates that 90 to 100 surgical fires occur annually in the United States [3]. More recently, the Joint Commission published an alert in October 2023 encouraging the implementation of preoperative risk assessments, fire prevention training, judicious use of supplemental oxygen and cautery, and periodic evaluation of operating room fire hazards [4]. In addition to the harm caused to the patient and operating room staff, intraoperative fires incur significant financial costs. The Ophthalmic Mutual Insurance Company (OMIC) reports significant postoperative complications in nearly half of all litigated intraoperative fires and a mean lawsuit settlement of $145,285 per case [5].

There are three components that must be present for a surgical fire to occur: an oxygen source, an ignition source (e.g. cautery, laser), and fuel (e.g. alcohol, aerosols, disposable drapes, towels, and, most importantly, human tissues). These three elements comprise what is colloquially known as the fire triangle. An oxygen-rich environment permits organic materials to sustain a fire they would otherwise not be able to in ambient air. Experimental studies of fires in oxygen-rich environments have demonstrated a direct correlation between ambient oxygen concentration and speed to combustion [6]. Moreover, ignition of volatile gases, fuel flanking the open end of a nasal cannula, or even the nasal cannula itself, may kickstart a blowtorch effect in which intraoperative fires can concentrate and cause focal injury [7]. Facial surgery regularly introduces a fire triangle since surgical procedures are often performed with electrosurgical or laser devices in the vicinity of nasal cannulated oxygen.

Current recommendations to minimize fire risk in facial surgery involve turning off oxygen supplementation and allowing local oxygen to dissipate for at least one minute before proceeding with cautery. These recommendations are not uniformly implemented because they require extensive coordination between the anesthetist and the surgeon who are often focused on their respective tasks. Moreover, intraoperative bleeding may be profuse or abrupt, prompting immediate cautery.

The authors previously demonstrated that an off-the-shelf medical grade midfacial seal drape effectively reduces surgical field oxygen concentration and may reduce the risk of intraoperative fire during oculofacial procedures [8]. An effective oxygen scavenger mask device may theoretically further reduce intraoperative fire risk and obviate the risks of dependent oxygen pooling in voids between facial curvatures or a sheet drape covering the entire middle and lower face. Additionally, a scavenger mask over only the oronasal region may reduce fire risk in surgeries involving the lower face and neck, including sites where a seal drape across the upper midface would not provide safety [8]. The authors herein test the efficacy of an oxygen scavenger mask prototype in reducing oxygen concentrations from nasal cannulated oxygen at various facial locations.

## Methods

### Oxygen scavenger mask prototype

Four customized oxygen scavenger masks with different size specifications and ventilation designs were designed and fabricated with fire-retardant silicone under U.S. Patent #10744286B2. Briefly, mask design molds were modeled with three-dimensional computer-aided design software (SolidWorks, Waltham, United States) and printed through a PolyJet 3D Printer (Stratasys, Eden Prairie, United States). Pourable room-temperature silicone polymers were mixed and poured into printed molds and cured over several hours at room temperature before deflashing. Each mask was then constructed from two custom-molded silicone parts with off-the-shelf tubing before final assembly with silicone adhesive. All final mask designs consisted of a nasal cannula port and manifold connected to wall vacuum (Supplemental Fig. 1). The mask design that best fit an average adult human face was selected for experimental measurements as follows.

### Experimental measurements

**Fig. 1:**
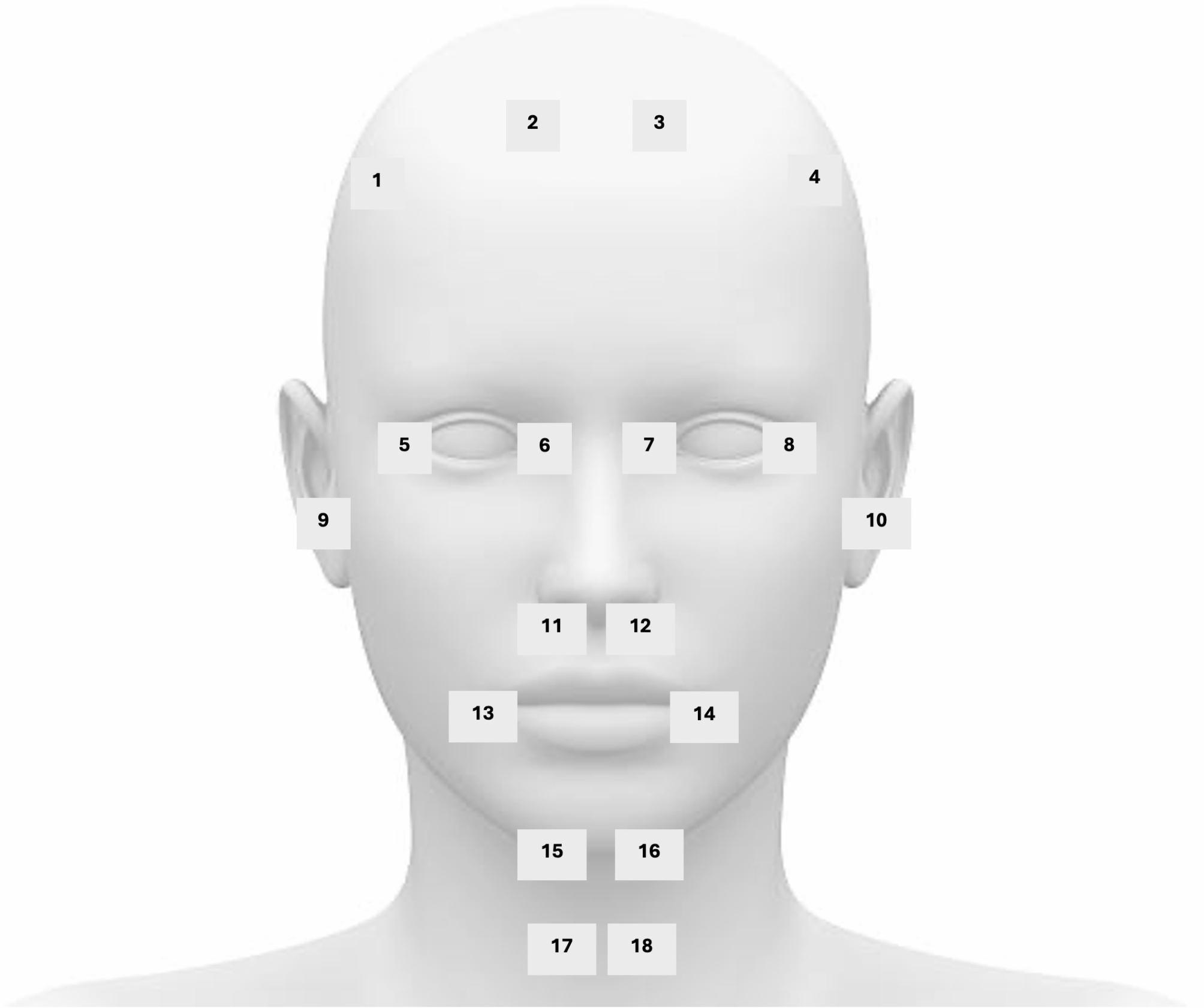
Diagram of facial landmarks assessed in this study

**Fig. 2:**
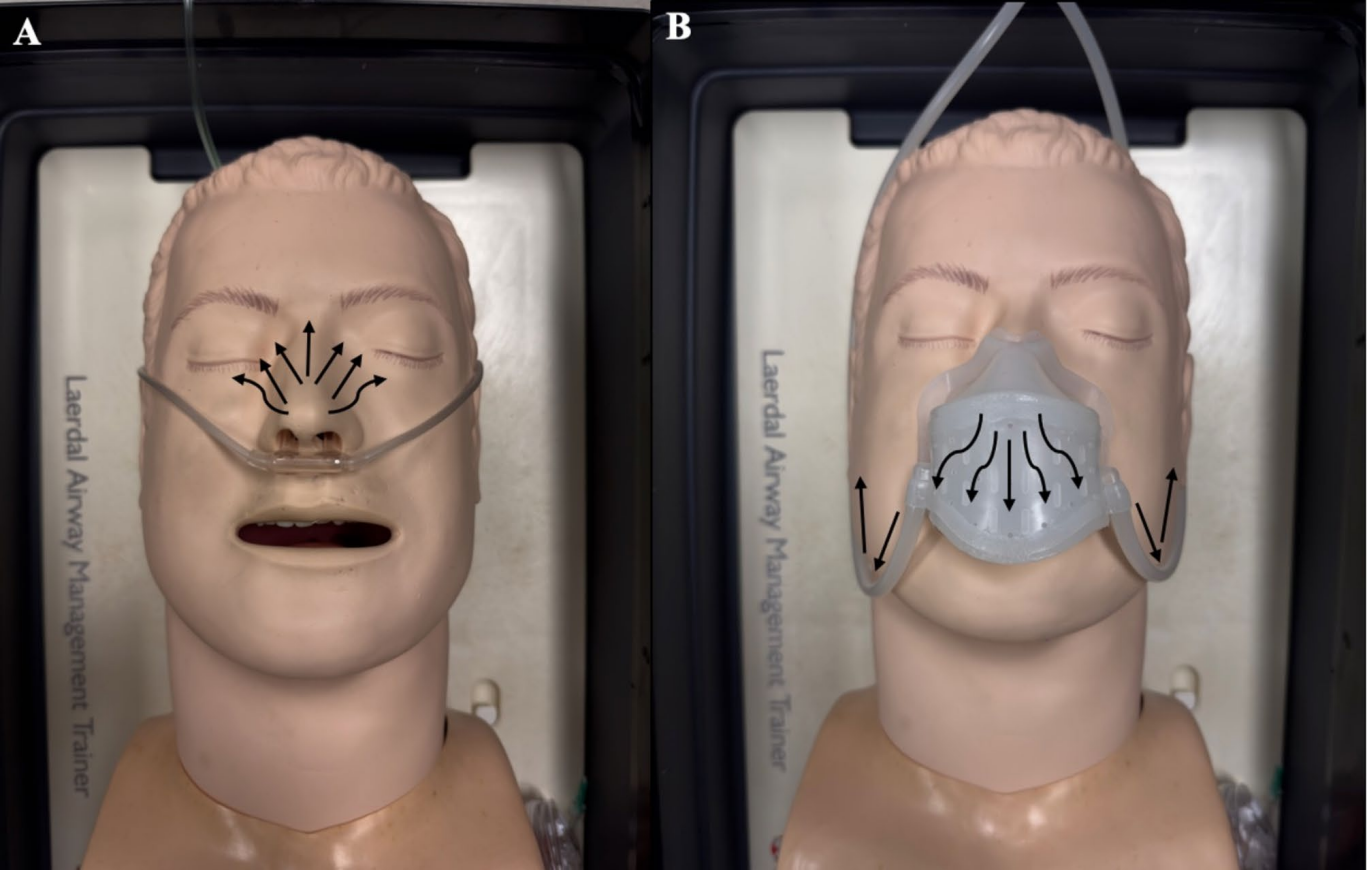
Representative photographs of the SimMan manikin in (**A**) unmasked and (**B**) masked conditions. Black arrows denote direction of oxygen flow

Experiments were conducted at the University of California, Irvine, Gavin Herbert Eye Institute using a supine SimMan^®^ airway management trainer manikin (Laerdal Medical, Stavanger, Norway). 100% FiO2 was delivered to the oculofacial field with nasal cannula flow rates of 2, 4, and 6 L/min using a Datex Ohmeda Aisys CarestationⓇ anesthesia unit (GE HealthCare, Chicago, United States) and wall vacuum was set to low (Ohio Medical, Gurnee, United States). End tidal ports designed to measure exhaled carbon dioxide were not used in this study. After allowing five minutes for equilibration between each flow rate, oxygen concentrations were measured at 18 facial landmarks (Fig. 1) using a handheld oxygen detector (Forensics DetectorsⓇ, Los Angeles, United States). Of note, oxygen concentrations at landmarks 11 through 16 were measured external to the mask. The oxygen sensor was placed perpendicular to the presumed direction of oxygen flow one inch from the face. Oxygen measurements were then repeated using the customized oxygen scavenger mask (Fig. 2). A total of three experimental replicates were performed.

### Statistical analysis

Local oxygen concentration with and without the device were compared via paired *t*-test at each nasal cannula flow rate. An oxygen concentration of 23% was our established threshold for intraoperative fire risk according to a previously published study [9].

## Results

The difference in mean oxygen concentration between the unmasked and masked conditions was statistically significant. Mean oxygen concentration in the facial surgical field without the oxygen scavenger mask was 24.8% ± 2.66%, while mean oxygen concentration with the device was 20.95% ± 0.34% (*P* < 0.001; two-tailed paired t-test). The scavenger mask device significantly reduced local oxygen concentration at 16 of 18 facial landmarks when compared to measurements recorded without it (Table 1). Without the device, suprathreshold oxygen concentration levels (i.e. 23% or greater) were seen at 13 of 18 facial landmarks, while the device provided subthreshold oxygen concentration levels (i.e. less than 23%) at all three flow rates.

**Table 1:** Average unmasked and masked oxygen concentrations. Each numerical value represents the average of oxygen concentrations measured at three flow rates. Italicized *P*-values denote statistical significance

Landmark	Average Unmasked Oxygen Concentration (%)	Average Masked Oxygen Concentration (%)	*P*-value
1	22.08	20.9	0.1799
2	22.44	20.9	0.0071**
3	22.37	20.92	0.0212*
4	21.96	20.9	0.2202
5	23.49	20.97	0.0252*
6	23.32	20.9	0.0168*
7	23.36	20.9	0.0173*
8	23.29	21	0.0477*
9	23.44	20.91	0.0269*
10	23.58	20.91	0.0361*
11	25.94	21.04	0.0003***
12	26.97	21.08	0.0025***
13	26.12	21.19	0.0001***
14	26.3	21.22	0.00003***
15	24.89	21.42	0.0008***
16	24.7	21.36	0.0019**
17	23.02	21.18	0.0266*
18	22.84	21.21	0.0252*

*P < 0.05, **P < 0.01, ***P < 0.001

The nares, mouth, and chin exhibited the highest suprathreshold local oxygen concentrations in the unmasked condition (Table 1). The periocular and preauricular regions also exhibited suprathreshold oxygen concentrations without the device, but to a lesser extent. The forehead, scalp, and neck exhibited subthreshold oxygen concentrations without the device. Oxygen concentrations at all facial locations were subthreshold at all facial landmarks with use of the device (Table 1).

Surgical field oxygen concentration directly correlated with oxygen flow rate in the unmasked condition at all facial landmarks (Table 2), with the nares, mouth, and chin exhibiting the strongest positive correlation (Table 2). Oxygen concentration measurements taken with use of the device did not exhibit the same trend and remained stable with increasing oxygen flow rate at all facial landmarks (Table 2).

**Table 2:** Average unmasked and masked oxygen concentrations at different oxygen flow rates. Each numerical value represents the average of three technical replicates. Italicized *P*-values denote statistical significance

Landmark	Flow Rate (L/min)	Average Unmasked Oxygen Concentration (%)	Average Masked Oxygen Concentration (%)	*P* value
1	2	20.9	20.9	N/A
	4	21.47	20.9	0.423
	6	23.87	20.9	0.312
2	2	22.47	20.9	0.041*
	4	21.73	20.9	0.253
	6	23.13	20.9	0.194
3	2	22.37	20.9	0.096
	4	21.33	20.97	0.257
	6	23.4	20.9	0.189
4	2	20.9	20.9	N/A
	4	21.37	20.9	0.331
	6	23.6	20.9	0.363
5	2	21.17	20.9	0.423
	4	23.2	21.1	0.108
	6	26.1	20.9	0.103
6	2	21.2	20.9	0.139
	4	23.83	20.9	0.113
	6	24.93	20.9	0.423
7	2	21.23	20.9	0.423
	4	23.97	20.9	0.127
	6	24.87	20.9	0.147
8	2	21.1	20.9	0.423
	4	23.17	21.2	0.121
	6	25.6	20.9	0.19
9	2	20.9	20.9	N/A
	4	24.1	20.93	0.142
	6	25.33	20.9	0.142
10	2	20.9	20.9	N/A
	4	24.07	20.93	0.124
	6	25.77	20.9	0.184
11	2	24.6	20.9	0.197
	4	26.73	21.33	0.050*
	6	26.5	20.9	0.033*
12	2	24.57	20.9	0.202
	4	26.8	21.43	0.049*
	6	26.53	20.9	0.026*
13	2	24.7	20.9	0.107
	4	27.5	21.77	0.033*
	6	26.17	20.9	0.063
14	2	25.47	20.9	0.089
	4	27.1	21.87	0.032*
	6	26.33	20.9	0.043*
15	2	23.5	20.9	0.112
	4	25.7	21.93	0.133
	6	25.47	21.43	0.078
16	2	23.17	20.9	0.115
	4	25.67	21.97	0.163
	6	25.27	21.2	0.103
17	2	22.6	21.5	0.16
	4	24.53	21.13	0.16
	6	21.93	20.9	0.423
18	2	22.33	21.2	0.083
	4	24.53	21.53	0.174
	6	21.67	20.9	0.423

*P < 0.05, **P < 0.01, ***P < 0.001

## Discussion

In this study, the authors find that a custom-designed oxygen scavenger mask reduces local oxygen concentrations at 18 distinct landmarks across the facial surgical field including the non-perinasal lower face to below the fire threshold on a simulated manikin model. These oxygen concentration reductions were statistically significant at all measured facial landmarks in the masked condition compared a standard unmasked condition. The oxygen concentration measurements were below fire threshold in the masked condition at all three tested flow rates. Oxygen concentration directly correlated with oxygen flow rate in the unmasked condition but not with the mask device, suggesting that an oxygen scavenger mask system may be a useful adjunct for reducing intraoperative fire risk. These findings build on a previous report describing the efficacy of a midfacial seal drape at diverting oxygen from the oculofacial surgical field [8]. An oxygen scavenger mask device may also obviate the limitations of a seal drape for lower facial surgery in reducing intraoperative fire risk.

Previous reports have noted that surgical oxygen levels create a fire hazard when concentration is at or above 23% [9]. In this study, landmarks near the nares and mouth displayed local oxygen concentrations above the fire threshold even at the lowest oxygen flow rate (2 L/min). Landmarks near the eye and neck regions displayed suprathreshold oxygen concentrations at a moderate flow rate (4 L/min), while the forehead and neck only displayed suprathreshold oxygen concentrations at a high flow rate (6 L/min). This pattern of oxygen distribution is likely secondary to the dependent pooling of oxygen. Oxygen is denser than air and, in our experiment, the most dependent parts of the face (i.e. nose and mouth) exhibited the highest local oxygen concentrations at the lowest flow rates [10].

A fire triangle during oculofacial surgery is unavoidable if a patient requires oxygen supplementation and cautery. The prototypes tested this study were constructed from inert silicone that may further add fire-retardant benefit. Currently available drape materials and masks are often constructed from organic materials and therefore can fuel a surgical fire. The chief feature of the prototypes, however, may be their ability to divert pooled oxygen from the operative field. Such a scavenger mask may minimize, but not eliminate, the risk of surgical fire. Further experiments with scavenger devices constructed from other materials are warranted.

Other approaches to surgical fire reduction, such as the use of nonflammable linens or drapes and minimizing or turning off supplemental oxygen prior to cautery, remain valuable. Awareness and understanding of how operating room fires occur are critical. Of note, smoke scavenger devices connected to electrosurgical cautery pens currently exist, but may exacerbate fire risk by drawing oxygen toward the ignition source. That hundreds of operating room fires continue to occur each year in the U.S. corroborates an ongoing need for further thoughtfulness, innovation, and education of fire hazards in the operating room.

One limitation of this study is that it was performed on an anesthesia training manikin that does not recapitulate all aspects of live human surgery. First, the seal of our scavenger mask may be more robust on the synthetic rubber surface of the manikin compared to surgically-prepared human skin, which is usually layered with antiseptic preparation solutions, natural oils and secretions, and condensed breath that may impair the formation of an adequate seal. Additionally, our mask designs are imperfect. They feed and scavenge oxygen in the same space, which may impair oxygenation in a live patient. Their bulkiness may also limit functionality in live human surgery by distorting facial anatomy and possibly interfere with facial surgery. Second, the manikin is immobile and lacks the head movements or breathing patterns of live patients under monitored anesthesia care. Inspiration and exhalation could affect local oxygen concentrations by either drawing oxygen into the airway or blowing oxygen away from the face. Third, facial anatomy varies on a patient-to-patient basis and may deviate from that of the manikin used in this study. We note a paradoxical reduction in oxygen concentration at the 6 L/min flow rate at landmarks 17 and 18, which would not be expected in a live patient. This may be secondary to the fluid dynamic system established by the facial structure of the manikin used in this study. As such, the oxygen distribution patterns observed in this study may differ from those seen in live patients. Formal experiments in human subjects may address many of these limitations and warrant further consideration.

In summary, an oxygen scavenger mask may offer additional protection against intraoperative fire during ambulatory facial surgery. This innovation, along with other pre-existing fire-retardant protocols, may enhance patient fire safety. Further studies that validate these theoretical safety improvements in clinical practice may be useful. Also, ergonomic design modifications that minimize bulk and area of facial coverage that offer optimal comfort and patient fit may be warranted.

## Electronic supplementary material

Below is the link to the electronic supplementary material.

Supplemental Figure 1: Representative image of prototype mask ports

## Data Availability

No datasets were generated or analysed during the current study.
