# Supplementary figures and images for: Efficacy of a novel oxygen scavenger mask in reducing local oxygen concentrations below the surgical fire risk threshold: an experimental proof-of-concept study
Source: Patient Saf Surg. 2024 Sep 12;18:27. doi: 10.1186/s13037-024-00411-1 (PMC11391775; doi:10.1186/s13037-024-00411-1)

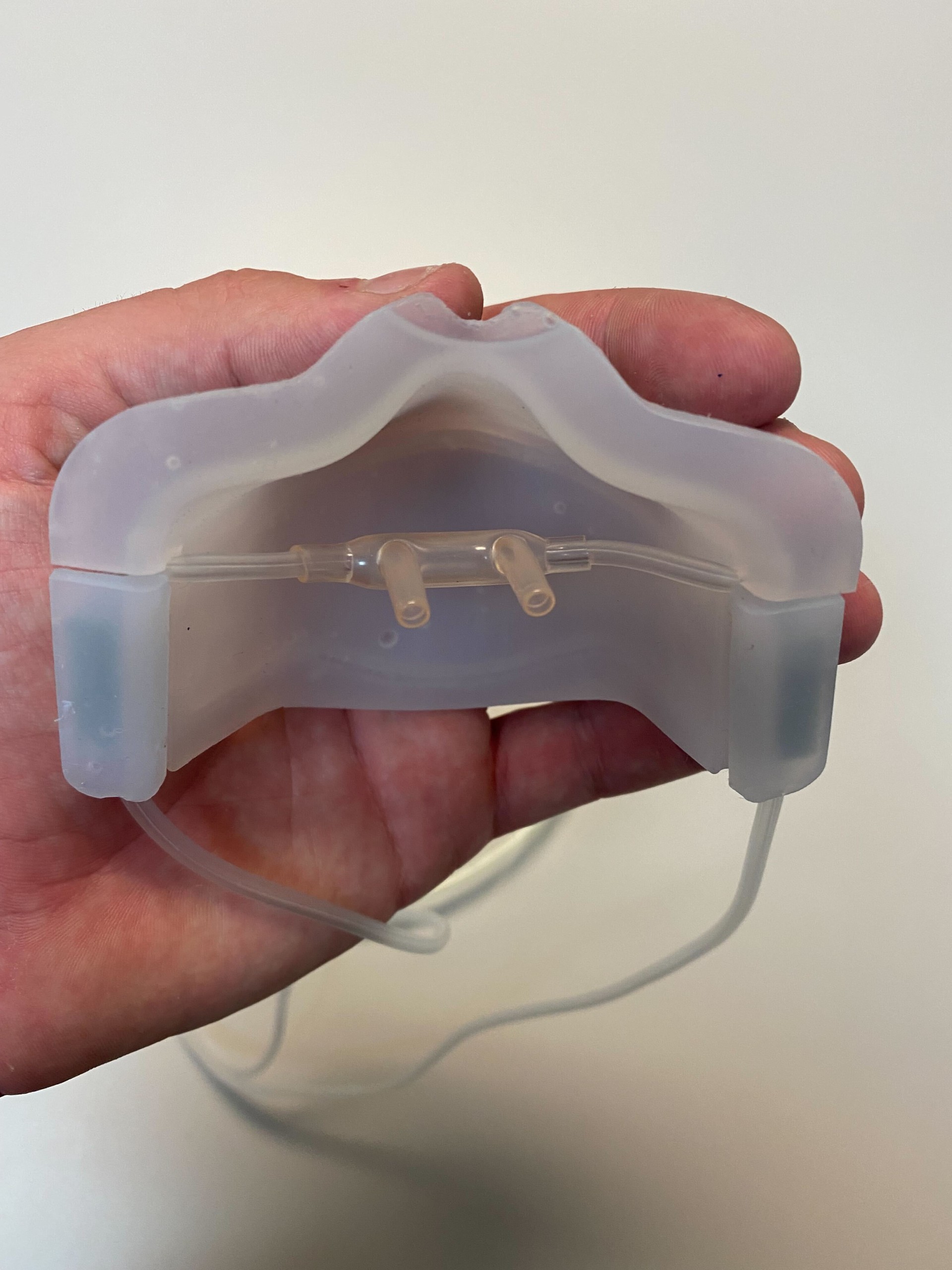

Supplement: Supplementary file 1 — Supplemental Figure 1: Representative image of prototype mask ports [file 13037_2024_411_MOESM1_ESM.jpg]
